# Pediatric Microspherophakia Treatment With Scleral Fixated IOL Using a Z-suture Technique

**DOI:** 10.7759/cureus.50445

**Published:** 2023-12-13

**Authors:** Renato Correia Barbosa, Rui Carvalho

**Affiliations:** 1 Ophthalmology, Hospital Pedro Hispano - Unidade Local de Saúde de Matosinhos (ULSM), Matosinhos, PRT

**Keywords:** z-suture scleral fixation, scleral fixation of intraocular lenses, iol (intraocular lens), marfan's syndrome, microspherophakia

## Abstract

Microspherophakia constitutes a rare, mostly bilateral anomaly of the crystalline lens, which is characterized by the presence of an increased lens thickness and reduced equatorial diameter. It is frequently associated with lens subluxation, translating into a high degree of variable lenticular myopia and defective accommodation. The purpose of this report is to describe the treatment of a three-year-old female patient with microspherophakia, with the scleral fixation of an intraocular lens using the z-suture technique.

A three-year-old female patient with Marfan Syndrome presented with high bilateral myopia and esotropia. Lens subluxation was perceived, and she was proposed for bilateral surgery. Scleral fixation of the intraocular lens was performed using the z-suture technique. During the five-year follow-up period, she maintained a best-corrected visual acuity of 20/20 in both eyes wearing bifocal glasses.

Microspherophakia is a rare but impactful condition, frequently related to severe and variable refractive error due to the lens shape and zonule instability. Intraocular lens implantation in the capsular bag is usually impossible, and scleral fixation is a valid alternative. The z-suture technique avoids suture knots and the need for intrascleral flaps, reducing the risk of suture-related complications.

## Introduction

Microspherophakia constitutes a rare, mostly bilateral anomaly of the crystalline lens, which is characterized by the presence of an increased lens thickness and reduced equatorial diameter. Its prevalence is not entirely established, and it is associated with several ocular and systemic diseases, including Marfan syndrome, Weill-Marchesani syndrome, Alport syndrome, and homocystinuria. The lens abnormality is frequently associated with subluxation, translating into a high degree of variable lenticular myopia and defective accommodation. Angle-closure glaucoma is a frequent associated complication and may be the initial presentation [1,2].

Scleral fixation of intraocular lenses is a surgical option in cases of washout of adequate capsular support or zonular insufficiency [3]. The z-suture technique, introduced by Szurman, is a knotless technique for transscleral suture fixation of IOLs, in which a needle is passed through the ciliary sulcus, and the suture is secured in the sclera by a zig-zag-shaped intrascleral suture, which is then cut without any knot [4].

The purpose of this report is to describe the case of a three-year-old female patient with Marfan syndrome, who was diagnosed with microspherophakia resulting in lens subluxation and severe zonular instability. She was treated with the fixation of an intraocular lens scleral fixation using the z-suture technique. The clinical case shown in this article was previously presented as an e-poster at the 26th European Society of Cataract and Refractive Surgeons Winter meeting, in February 2022.

## Case presentation

**Figure 1:**
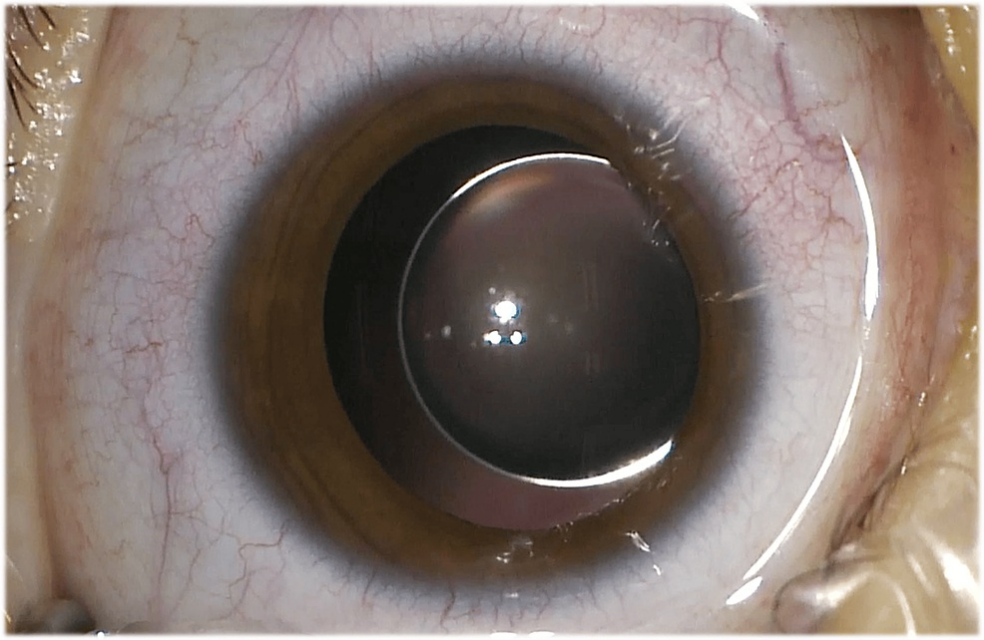
Photograph showing the presence of microspherophakia with subluxation of the crystalline lens

A three-year-old female patient with a direct familiar history of Marfan syndrome presented with very high bilateral myopia of -20.00D, objectified by retinoscopy, and variable-angle alternating esotropia. Microspherophakia with lens subluxation was perceived, along with an extremely variable refractive error, which was dependent on the child’s postural position. Given the impossibility of refractive correction and the risk of long-term amblyopia, she was proposed for bilateral crystalline lens surgery. The power of the IOL was calculated based on optical biometry, which the patient, despite her age, was able to perform. The target refraction was aimed at emmetropia. During surgery, severe zonular instability with practically total detachment of the lens capsular bag was objectified (Figure 1).

**Figure 2:**
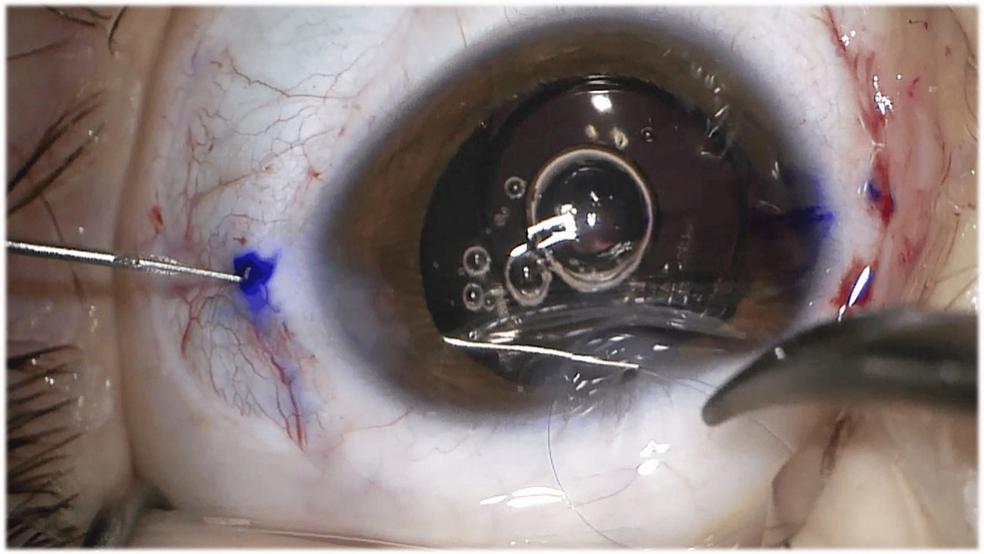
Surgical step: The needle was passed behind the iris and externalized through the sclera at a distance of 2 mm from the limbus, guided by a bent 27-gauge insulin injector needle

**Figure 3:**
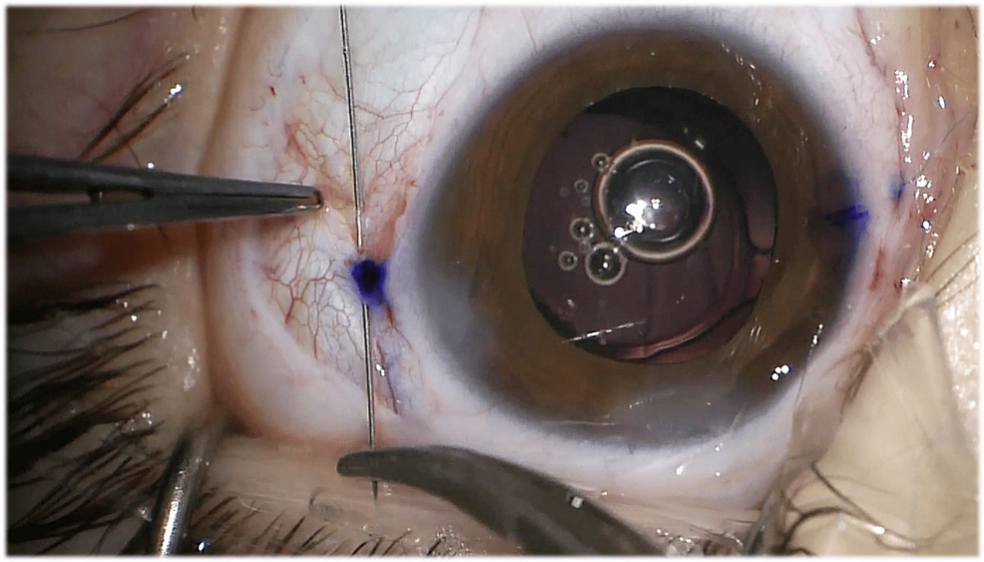
Surgical step: Instrascleral suture passes made parallel to the limbus

Therefore, given the impossibility of stabilizing the capsular bag and implanting an IOL inside it, it was decided to perform a lensectomy with scleral fixation of the IOL. After the aspiration of the crystalline lens and anterior vitrectomy with complete removal of the capsular bag, a conjunctival peritomy was performed at the 3 o’clock and 9 o’clock positions along the limbus to expose the underlying sclera. The anterior chamber was filled with OVD, and the IOL (Akreos AO® (Bausch & Lomb, New York)) was implanted. Then, one of the haptics was externalized with forceps, and a straight needle 10-0 polypropylene suture was passed through its eyelet, forming a loop, which was sutured to the haptic. This process was repeated twice, and the needle was inserted inside the eye through the corneal incision. The needle was then passed behind the iris and externalized through the sclera at a distance of 2 mm from the limbus, guided by a bent 27-gauge insulin injector needle, at a 3 or 9 o’clock position. Two intrascleral passes, approximately 5 mm long, were then made parallel to the limbus. The procedure was repeated for the opposite haptic, which was fixed to the opposite position, 180° away from the initial location, to ensure correct positioning and centration of the IOL. After two intrascleral passes were made on each side, three additional passes were made bilaterally, for a total of five intrascleral passes on each side, which is the recommended number to resist the maximum tensile force of 0,41 Newton, the most that the suture itself can withstand [4]. Finally, the sutures are cut adjacent to the sclera, without knotting. The conjunctiva was then closed with polyglactin (Vicryl®) 8-0 suture, the OVD was removed, and the corneal incisions were sealed by hydrating the stroma. Subconjunctival injections of cefuroxime and methylprednisolone were administered. Figures 2, 3 show the main surgical steps of the surgical procedure of the second eye.

**Figure 4:**
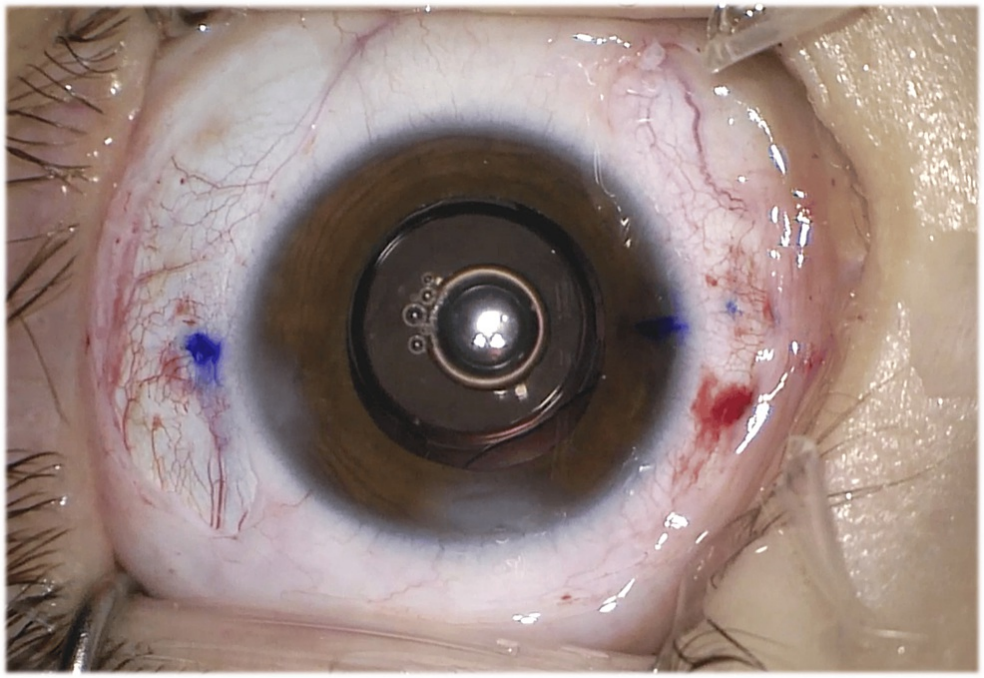
Centration of the intraocular lens in the posterior chamber at the end of the surgery

At the end of the surgery, proper centration of the intraocular lens in the posterior chamber was achieved (Figure 4).

During the five-year follow-up period, the patient had a best-corrected visual acuity (BCVA) of 20/20 in both eyes, wearing bifocal glasses. There were no complications such as decentration or subluxation of the implanted IOL.

## Discussion

Microspherophakia is an uncommon but severe condition, usually related to genetic syndromes that affect protein synthesis. Most cases are associated with severe zonular instability, preventing the implantation of the IOL inside the capsular bag [2,3].

Scleral fixation of the IOL may constitute a valid alternative for those cases. It constitutes a safe option, which places the IOL in a position close to the natural lens plane, and theoretically avoid drawbacks associated with other type of aphakic lenses. The most common techniques require a knot, which in the long term may be related to consequences related to the exposure of the underlying sclera, and to the pathway which is created by the suture thread itself to the interior of the eyeball, raising the risk of infection [5-7]. The z-suture technique enables the creation of a transscleral fixation without the need for a knot, preventing direct contact with the conjunctiva in its trajectory, which avoids irregularities in the scleral surface responsible for erosion [4]. The Akreos AO® (Bausch & Lomb, New York) IOL was the chosen lens because its design with four haptics enables adequate support for the sutures. A potential disadvantage of this technique is the fact that only two of the four haptics are used to fixate the IOL, which may raise concerns about the long-term stability of the lens, especially regarding the risk of tilting [8,9].

The final BCVA was 20/20 in both eyes, which was a very satisfactory functional result, especially knowing that previous fluctuating high myopia, due to the lens subluxation made the use of refractive correction incompatible and led to very low visual acuities. This was a particularly serious problem, given the patient’s age, and the risk of amblyopia and functional impairment, particularly in terms of school performance and the ability to learn fundamental skills, such as reading and writing.

## Conclusions

Microspherophakia is usually related to high lenticular myopia due to altered lens shape and zonular instability. Its surgical treatment is challenging, given the fact that a conventional IOL implantation in the capsular bag is usually not possible. Scleral fixation of the intraocular lens is a therapeutic alternative, and the z-suture technique avoids long-term complications regarding the suture knots while providing lens centration and stability.
